# Fructose Causes Liver Damage, Polyploidy, and Dysplasia in the Setting of Short Telomeres and p53 Loss

**DOI:** 10.3390/metabo11060394

**Published:** 2021-06-17

**Authors:** Christopher Chronowski, Viktor Akhanov, Doug Chan, Andre Catic, Milton Finegold, Ergün Sahin

**Affiliations:** 1 Huffington Center on Aging, Baylor College of Medicine, Houston, TX 77030, USA; christopher.chronowski@bcm.edu (C.C.); Viktor.Akhanov@bcm.edu (V.A.); Andre.Catic@bcm.edu (A.C.); 2 Department of Molecular and Cellular Biology, Baylor College of Medicine, Houston, TX 77030, USA; dchan@bcm.edu; 3 Department of Pathology, Baylor College of Medicine, Houston, TX 77030, USA; finegold@bcm.edu; 4 Department of Physiology and Biophysics, Baylor College of Medicine, Houston, TX 77030, USA

**Keywords:** telomeres, liver fibrosis, p53, fructose, triglyceride

## Abstract

Studies in humans and model systems have established an important role of short telomeres in predisposing to liver fibrosis through pathways that are incompletely understood. Recent studies have shown that telomere dysfunction impairs cellular metabolism, but whether and how these metabolic alterations contribute to liver fibrosis is not well understood. Here, we investigated whether short telomeres change the hepatic response to metabolic stress induced by fructose, a sugar that is highly implicated in non-alcoholic fatty liver disease. We find that telomere shortening in telomerase knockout mice (TKO) imparts a pronounced susceptibility to fructose as reflected in the activation of p53, increased apoptosis, and senescence, despite lower hepatic fat accumulation in TKO mice compared to wild type mice with long telomeres. The decreased fat accumulation in TKO is mediated by p53 and deletion of p53 normalizes hepatic fat content but also causes polyploidy, polynuclearization, dysplasia, cell death, and liver damage. Together, these studies suggest that liver tissue with short telomers are highly susceptible to fructose and respond with p53 activation and liver damage that is further exacerbated when p53 is lost resulting in dysplastic changes.

## 1. Introduction

Telomeres are the repetitive ends of chromosomes that are synthesized by the dedicated enzyme telomerase [1,2,3,4]. This multicomplex enzyme consists of a reverse transcriptase component (TERT), a RNA template (TERC), and associated proteins and prevents telomere attrition by adding telomeric TTAGGG repeats to chromosome ends [2,4,5]. In humans, telomerase is turned off in the majority of somatic cells but present in stem and progenitor cells, albeit at levels insufficient to maintain telomere length over a lifetime [6,7]. Advanced telomere shortening is recognized as DNA damage by the DNA damage surveillance machinery that activates the classical p53-mediated checkpoint response of growth arrest, senescence, and apoptosis [8,9,10,11,12]. While these cellular responses are potent barriers for tumorigenesis, they are also implicated in driving a variety of disorders in patients with critical short telomeres due to loss-of function mutations of telomerase (telomere biology disorders, TBD) and in the aged [13,14,15]. Accelerated telomere shortening is also observed in disorders associated with high cell turnover (such as inflammatory bowel disease or hepatitis virus infection) and thought to contribute to disease progression and complications in these chronic conditions [16,17,18,19].

In TBD patients, stem cell-dependent tissues such as the hematopoietic system and skin are most prominently affected, indicating that the activation of p53 and elimination of stem and progenitor cells is an important pathogenic mechanism [13,20,21,22]. This is supported by the amelioration of regeneration in stem cell-dependent tissues when p53 or its downstream targets are deleted [11,23,24,25]. Interestingly, in a fraction of these patients, tissues with low proliferative index such as the lung and the liver are affected by fibrosis, a process characterized by continuous parenchymal cell death and deposition of excess amounts of extracellular matrix [14,26,27,28]. How short telomeres predispose TBD patients to liver fibrosis is not well understood [29]. Specifically, the instigating factors and underlying mechanisms that cause continuous cell death in the liver are not known.

The liver, as the central organ for systemic metabolism, is a highly active metabolic organ that processes nutrients, carries out detoxification, and regulates blood glucose and lipids levels, among other functions. In the Western world in particular, excess food consumption has increased the prevalence of non-alcoholic fatty liver disease (NAFLD), a spectrum of disorders that are characterized by the accumulation of excess fat and that can range from simple fatty liver (steatosis) to inflammation (nonalcoholic steatohepatitis, NASH). It is estimated that 25% and 5% of the US population has NAFLD and NASH, respectively. NASH is a condition that can lead to complications, including cirrhosis, liver transplantation-requiring end stage liver failure, and hepatocellular carcinoma [30,31]. Among the various dietary factors implicated in the NAFLD pathogenesis, increased fructose consumption has garnered specific attention as an important driver due to a large body of studies in humans and rodents [32,33,34,35,36,37]. Fructose is thought to be particularly toxic, as it potently drives de-novo lipogenesis, depletes ATP, and induces reactive oxygen species [38,39].

Besides an improved understanding of how specific nutrients contribute to NAFLD, the identification of host-intrinsic factors that predispose to NAFLD and disease progression remains an important area of research. Here we hypothesized that hepatocytes with short telomeres are functionally compromised and susceptible to increased metabolic stress based on the recent discovery that telomere shortening compromises cellular metabolism through p53-dependent repression of sirtuins and peroxisome proliferator- activated receptor gamma co-activator 1 α/β (PGC-1α/β) [14,40]. These changes are associated with impaired mitochondrial biogenesis and function, resulting in decreased ATP synthesis and elevated ROS levels [40,41]. We reasoned that these short telomere-induced alterations could predispose hepatocytes to cell death, senescence, and fibrosis. Such a mechanism could not only be relevant for TBD patients but also for those with acquired conditions that are associated with short telomeres, including chronic hepatitis and fatty liver disease itself [42,43]. Our studies demonstrate that telomere shortening impairs the accumulation of TG in response to a fructose-rich diet in a p53-dependent manner. Despite this reduced fat content, hepatocytes with short telomeres are susceptible to fructose and undergo apoptosis and senescence. Deletion of p53 leads to TG accumulation, polyploidization, and dysplastic changes and further accelerates liver damage and fibrosis.

## 2. Results

To investigate whether telomere shortening impacts the response to increased fructose consumption, we utilized mice with short telomeres generated through successive interbreeding of mice deficient for the reverse transcriptase component of telomerase, TERT, as described previously [44]. The third generation of TERT null mice (hereafter referred to as “TKO”) displays hallmarks of short telomeres, including activation of the DNA damage response pathway and develops classical telomere shortening-associated pathologies, including regenerative defects in high turn-over tissues, tissue atrophy, predisposition to fibrosis, and shortened lifespan, as we have reported previously [40]. We used male TKO mice with age- and sex-matched wild type mice (WT) serving as normal telomere length controls.

**Figure 1:**
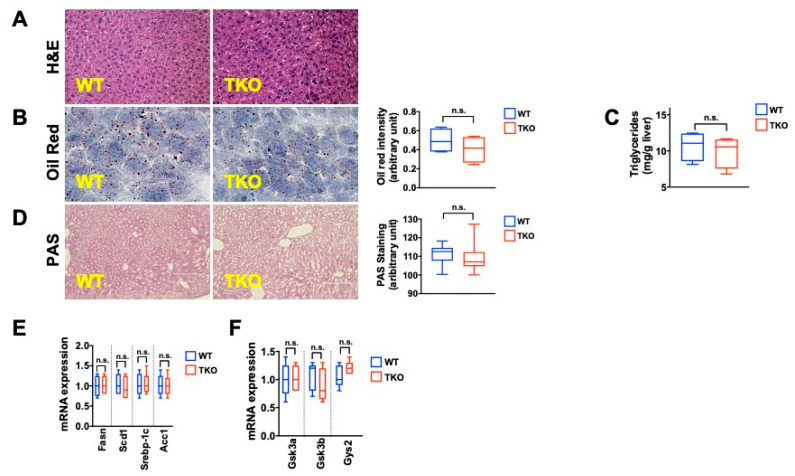
TKO are indistinguishable from WT mice on regular chow diet (**A**) H&E staining demonstrates normal liver architecture in TKO mice (**B**) Oil Red O (ORO) staining indicates similar triglyceride levels between TKO and WT mice (right graph shows quantification) (**C**) Biochemical TG quantification shows similar levels in WT and TKO mice (**D**) Periodic-Acid-Schiff (PAS) staining indicates similar glycogen content in WT and TKO mice (**E**) RT-qPCR demonstrates no differences in the expression of genes regulating TG biosynthesis (**F**) RT-qPCR demonstrates no differences in the expression of genes regulating glycogen biosynthesis. 5–8 mice were analyzed per group for each experiment and statistical differences were calculated by *t*-test. n.s. = not significant.

In the liver, TKO mice on regular chow (PicoLab, 5V5R) display normal liver architecture and contain similar triglyceride and glycogen levels compared to their WT counterparts, as assessed by Oil Red O (ORO), biochemical analysis and Periodic acid–Schiff (PAS) staining (Figure 1A–D; 5–8 mice per group analyzed). RT-qPCR analysis of key enzymes for triglyceride (SREBP-1c, FASN, SCD1, ACC1) and glycogen (GSK3a, GSK3b, GYS2) synthesis shows similar transcript levels in TKO and WT mice (Figure 1E,F; 5–8 mice analyzed per group). Liver integrity, measured by damage-indicating enzymes (ALT, AST, ALP), synthesis capacity (Albumin, Globulin), and fat metabolism (cholesterol, HDL, LDL, VLDL) were comparable between TKO and WT mice (Figure S1A–D; five mice per group analyzed), indicating that under baseline conditions telomere dysfunction does not compromise liver integrity or basic functions.

**Figure 2:**
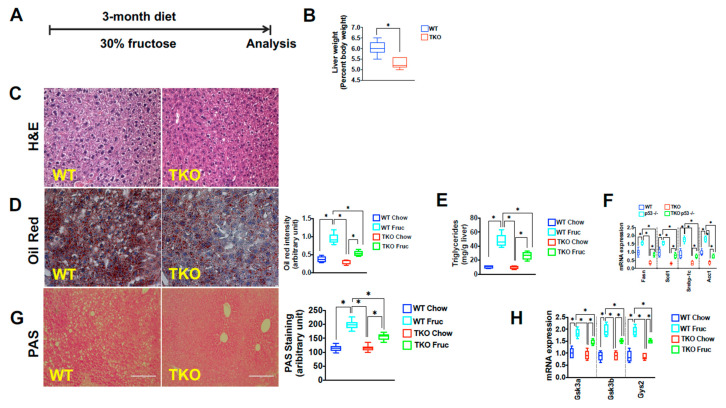
Mice with short telomeres accumulate less fat in liver tissue upon fructose treatment (**A**) Schematic fructose treatment regime and duration; (**B**) Fructose-treated TKO livers are smaller than WT livers (**C**) H&E staining shows fewer fat vacuoles in TKO liver tissue, indicating decreased fat content; (**D**) Oil Red O staining demonstrates reduced fat accumulation in TKO mice, the right graph shows quantification; (**E**) TKO mice have significantly less hepatic triglycerides (TG) as determined by biochemical analysis; (**F**) TKO mice have lower transcript levels of genes regulating TG synthesis based on RT-qPCR analysis; (**G**) PAS staining demonstrates decreased glycogen content in TKO mice; (**H**) TKO mice have lower transcript levels of genes regulating TG synthesis based on RT-qPCR analysis. 8–10 mice per group were analyzed for each experiment and statistical differences were calculated by *t*-test. * denotes *p* < 0.05.

**Figure 3:**
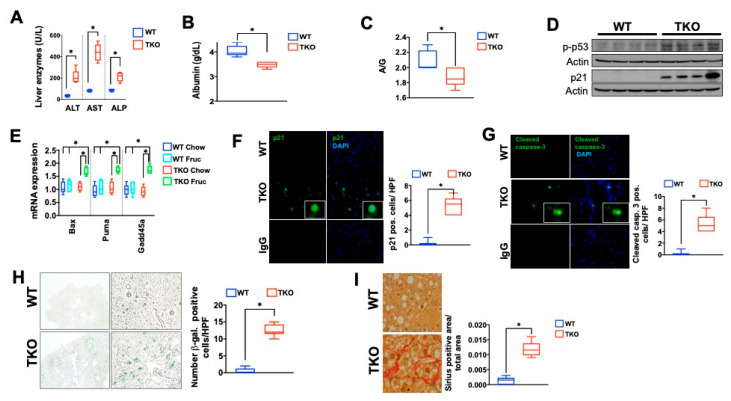
A fructose–rich diet induces DNA damage response, cellular damage, and senescence in mice with short telomeres. (**A**) Liver transaminases are increased in TKO mice, indicating hepatocyte damage; (**B**,**C**) Albumin concentration and Albumin/Globulin ratio are decreased as a sign of reduced hepatocyte synthesis capacity; (**D**) Phosphorylated p53 and total p21 protein are increased in TKO liver; (**E**) RT-qPCR analysis of p53 targets indicate significant increase of p21, Bax, Puma and Gadd45a; (**F**,**G**) TKO mice have increased expression of p21 and cleaved caspase 3 by immunofluorescence; (**H**) Hepatocyte senescence is a pronounced response in TKO mice on fructose diet; (**I**) TKO mice have increased collagen deposition detected by sirius red staining as sign of chronic damage and mild fibrosis. 6–10 mice per group were analyzed for each experiment and statistical differences were calculated by *t*-test. * denotes *p* < 0.05.

Recent studies have demonstrated that telomere dysfunction and the DNA damage response-associated activation of p53 converge on cellular metabolism by downregulating major regulators of cellular metabolism including Sirt1, PGC-1α, and PGC-1β [40,45,46]. The downregulation of these factors is associated with reduced mitochondrial biogenesis and function, as reflected by increased ROS levels and reduced ATP synthesis capacity, particularly in the face of liver damage induced by hepatotoxins [41]. To probe the functional relevance of these alterations under metabolic stress conditions, we exposed TKO and control WT mice to a high fructose diet reasoning that the increased demand to metabolize this sugar might uncover a functional impairment when telomeres are critically short. To this end, eight-week-old TKO and WT mice were exposed to 30% fructose and followed for three months (Figure 2A). Under these conditions, TKO mice were undisguisable from WT mice in their food and water intake as well as energy expenditure (Figure S2A–C, *n* = 5 per group). TKO livers were significantly smaller than their WT counterparts at the end of the study, indicating less fat accumulation (Figure 2B; *n*=10 per group; *p* < 0.05). Indeed, TKO mice accumulated significantly less fat in the liver as assessed by H&E, ORO, and biochemical analysis (Figure 2C–E; *n* = 10 mice; two independent experiments; *p* < 0.05). Compared to control WT mice, TKO mice displayed approximately 50% less hepatic TG accumulation (Figure 2D,E; *n* = 6 mice; *p* < 0.05). The reduced TG content in TKO mice was paralleled by a blunted expression of lipogenic genes including SREBP-1c, FASN, ACC1, and SCD1 compared to WT mice on a fructose diet (Figure 2F; *n* = 6; *p* < 0.05). A clear transcriptional response to fructose was noticeable in TKO mice as the expression of TG-synthesizing enzymes was increased compared to TKO mice on regular chow, although this response was blunted compared to WT mice on a fructose diet (Figure 2F; *n* = 6; *p* < 0.05). Similarly, the hepatic glycogen content was significantly reduced in TKO mice in response to fructose, which was associated with a decreased expression of the glycogenic enzymes Gsk3a, Gsk3b, and Gys2, compared to fructose-exposed WT mice (Figure 2G,H; *n* = 10 per group for PAS and *n* = 6 for RT-qPCR analysis; *p* < 0.05). To gauge the consequence of increased fructose consumption, we analyzed markers indicative of liver damage and synthesis capacity. TKO livers displayed significant susceptibility to damage and reduced synthesis capacity in response to fructose, as reflected by the increase of liver transaminases (Figure 3A; *n* = 6 per group; *p* < 0.05), lower blood protein, albumin levels, and decreased albumin/globulin ratio (Figure 3B,C; *n* = 6 per group; *p* < 0.05). Western blotting, RT-qPCR, and immunofluorescence analysis revealed that the hepatic damage in TKO mice was associated with increased expression of phosphorylated p53 and its downstream pro-apoptotic targets (Bax, Puma, Gadd45a; Figure 3D,E; *n* = 6 per group; *p* < 0.05) and cell cycle regulator p21 (Figure 3F; *n* = 3 per group; *p* < 0.05). The activation of the DNA damage response can lead to cell death and senescence and both are significantly increased in TKO livers, as determined by cleaved caspase 3, Tunel staining and β-galactosidase staining (Figure 3G,H and data not shown; *n* = 6 per group; *p* < 0.05). These changes in TKO liver tissue were accompanied by increased deposition of collagen, as determined by Sirius Red staining indicative of chronic damage and mild fibrosis (Figure 3I; *n* = 6 per group; *p* < 0.05). Together, these studies indicated that hepatocytes with dysfunctional telomeres are highly susceptible to fructose and respond with a pronounced activation of p53, cell death, and senescence.

**Figure 4:**
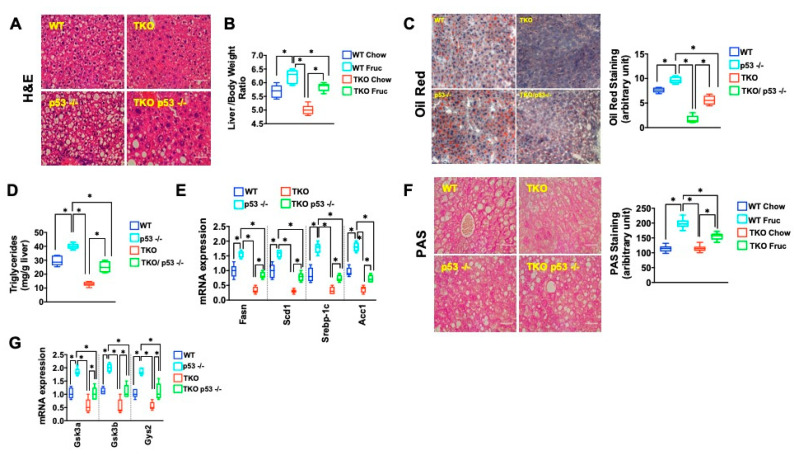
p53 deletion increases fat accumulation in TKO mice; (**A**) H&E shows significant increase of vacuoles in TKO mice that lack p53 indicating increased fat accumulation; (**B**) Liver /body weight ratio is increased in TKO/p53 −/− mice indicative of increased fat accumulation; (**C**) p53 deficiency in TKO mice increases fat accumulation as determined by ORO staining; (**D**) TG content in TKO livers lacking p53 is significantly increased compared to TKO mice with intact p53 (**E**) RT-qPCR analysis demonstrates increased expression of TG synthesis-regulating genes in TKO/p53 −/− mice (**F**) TKO/p53 −/− mice have increased glycogen compared to TKO p53 +/+ mice; right graph shows quantification (**G**) Expression of glycogen-synthesis genes is upregulated when p53 is deleted in TKO mice. Five mice per group were analyzed for each experiment and statistical differences were calculated by *t*-test.

We next wanted to understand the mechanism underlying this susceptibility and focused on the role of p53, which plays a central role in mediating telomere dysfunction-associated metabolic changes [40,41]. To this end, we generated TKO mice that were either proficient or deficient for p53 (TKO/p53 +/+ or TKO/p53 −/−) and used WT and p53 −/− mice as normal telomere controls. Baseline histology and blood chemistry analysis did not show any overt differences between the genotypes on a regular chow diet (data not shown). We then exposed mice of different genotypes to a fructose-rich diet and carried out indirect calorimetry using CLAMS (Comprehensive Lab Animal Monitoring System), which did not reveal any differences in oxygen consumption, carbon dioxide production, respiratory exchange ratio, total energy expenditure, and body temperature (data not shown). By H&E staining over 50%, hepatocytes in TKO/p53 −/− mice had large vacuoles that contrasted with the small vacuoles and in fewer hepatocytes (Figure 4A; *n* = 6 per group), which paralleled the heavier TKO/p53 −/− livers compared to TKO/p53 +/+ (Figure 4B; *n* = 6 per group; *p* < 0.05). ORO staining and TG measurement confirmed the significant difference in TG between TKO/ p53 −/− and TKO/p53 +/+ mice (Figure 4C,D; *n* = 6 per group; *p* < 0.05) and this was accompanied by increased levels of genes driving fat synthesis, indicating an important role of p53 in controlling fat metabolism in response to a fructose-rich diet (Figure 4E, *n* = 6 per group; *p* < 0.05). Interestingly, p53 deficiency in the setting of long telomeres increased TG accumulation as well, suggesting that p53 in the liver, under metabolic stress, represses fat synthesis, similar to what has been reported for p53 −/− that were exposed to a high fat diet (compare WT to p53 −/−; Figure 4A–D; *n* = 6 per group, *p* < 0.05) [45,46]. This was also indicated by the increased expression of key transcription factor Srebp1 and downstream targets FASN and enzyme ACC1 and SCD1 in p53 −/− (mice) compared to WT mice (Figure 4E; *n*= 6 per group, *p* < 0.05). Similar to TG accumulation, the glycogen content was affected by p53 status, as documented by the increase of glycogenic enzymes and glycogen in TKO p53 −/− mice (Figure 4G; *n* = 6 per group, *p* < 0.05).

**Figure 5:**
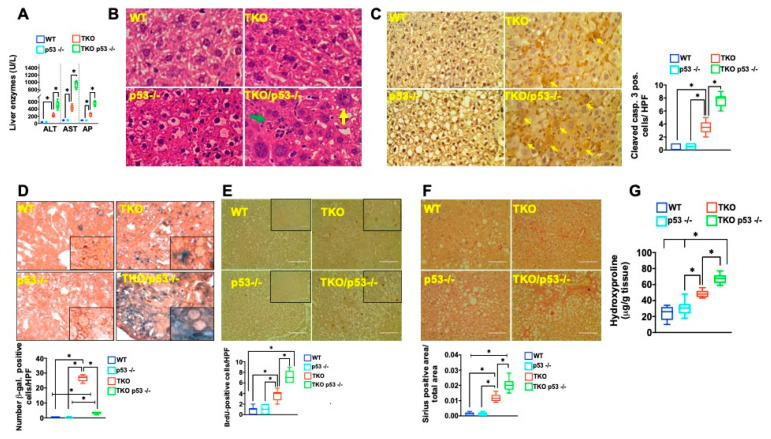
p53 deletion increases fat accumulation and damage in TKO mice (**A**) Liver transaminases (ALT, AST) and AP are significantly increased in TKO p53 −/− livers (**B**) H&E staining shows inflammation (green arrow) and dying hepatocytes (yellow arrow) (**C**) p53 deficiency in TKO mice increases cell death as measured cleaved caspase 3 staining (arrows point to cytoplasmatic staining of dying hepatocytes; right graph shows quantification (**D**) Senescence is mostly dependent on p53 as TKO p53 −/− have significantly fewer senescent cells compared to TKO p53 +/+ mice; graph beneath shows quantification (**E**) BrdU staining demonstrates an increase number of cycling hepatocytes in TKO p53 −/− mice; graph beneath depicts quantification (**F**,**G**) Increased Sirius red area and hydroxyproline concentration in TKO p53 −/− mice indicative of chronic damage and fibrosis. 5–8 mice per group were analyzed for each experiment and statistical differences were calculated by *t*-test. * denotes *p* < 0.05.

Next, we assessed the functional consequence of p53 deficiency and increased fat synthesis in the context of short and long telomeres. Interestingly, the improved metabolism upon p53 deletion caused further damage in TKO mice compared to TKO mice with intact p53. This was evident in the elevated levels of transaminases and decreased albumin/globulin ratio (Figure 5A and Figure S5A; *n* = 6 per group, *p* < 0.05). P53 deficiency also caused significant hepatocyte damage (disintegrating hepatocytes, yellow arrow) and inflammation (leukocytic infiltration, green arrow; Figure 5B; *n* = 6 per group). This was accompanied by increased apoptosis indicating the existence of p53-independent pathways that induce apoptosis (cleaved caspase 3, Figure 5C; *n* = 6 per group, *p* < 0.05). In contrast, the observed increase of senescence in TKO mice on a fructose diet was largely p53 dependent as TKO p53−/− mice showed very few senescent cells compared to TKO p53 +/+ mice (Figure 5D; *n* = 6 per group, *p* < 0.05). The increased damage was associated with increased proliferation (Figure 5E; *n* = 6 per group, *p* < 0.05) in TKO p53 −/− mice compared to their TKO p53 +/+ counterparts. The increased cell death and proliferation was paralleled by fibrosis in TKO p53 −/− mice as measured by Sirius red staining and hydroxyproline measurement (Figure 5F,G; *n* = 6 per group, *p* < 0.05). These changes were telomere length-dependent, as WT or p53 −/− with intact telomeres did not show any of these pathologies (Figure 5A–G; *n* = 6 per group, *p* < 0.05). Together, these studies showed that loss of p53 in telomere dysfunctional mice confers increased susceptibility to damage and leads to chronic damage and fibrosis in response to a fructose diet.

While the above studies indicate that p53 activation represses TG accumulation, the deletion of p53 at the whole-body level could lead to changes in other tissues that might account for the observed differences in the liver. To definitively answer whether p53 regulates fructose metabolism in a liver-specific manner, we took two complementary approaches. First, we used conditional p53 mice to delete p53 specifically in hepatocytes via the use of a liver specific Cre deletor line, AlbCre, in the context of either short (TKO/AlbCre/p53 fl/fl) or preserved telomere length (AlbCre/p53 fl/fl). As AlbCre is active during development and the results could be confounded by developmental adaptive processes, we took a second approach that relied on the acute deletion of p53 in adult TKO/p53 f/f and p53 fl/fl mice via injection of AAV-Cre. Both approaches yielded results that were comparable to those obtained in TKO that lacked p53 in all tissues. Deletion of p53 in TKO/AlbCre/p53 fl/fl caused a significant increase of TG and glycogen content accompanied by increased expression of genes driving TG and glycogen synthesis (Figure S5B–E; *n*= 6 per group, *p* < 0.05). Similarly, deletion of p53 in the liver of adult mice via AAV-Cre caused an increase of hepatic fat and glycogen content, demonstrating that lipid metabolism is under p53 control in a tissue and cell-specific manner (data not shown). Similar to TKO with whole body p53 deficiency, the deletion of p53 in the liver induced similar pathological changes including liver damage, hepatitis, cell death, proliferation, and fibrosis (data not shown).

**Figure 6:**
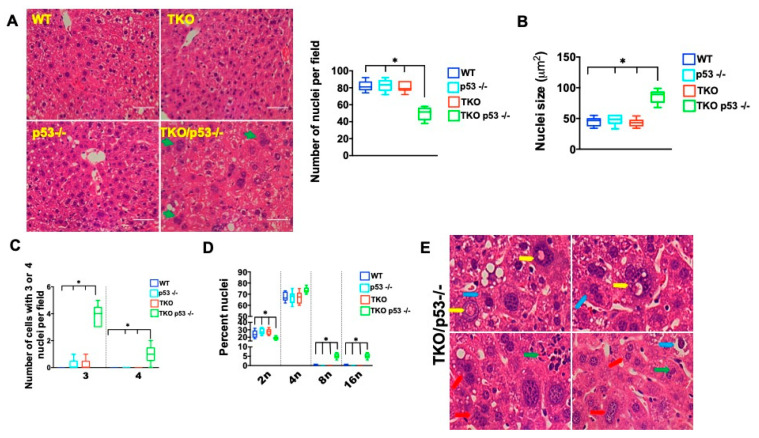
P53 deletion causes polyploidy, polynuclearization, and dysplastic changes in TKO mice on a fructose diet. (**A**) H&E staining demonstrates pronounced heterogeneity in cell size, the right graph shows quantification of cell numbers per field; (**B**) Nuclear size is significantly increased in TKO p53 −/− mice; (**C**) Number of cells with three or four nuclei is elevated in TKO p53 −/− mice; (**D**) FACS analysis demonstrates increased ploidy in TKO p53 −/− mice; (**E**) Different cytological and tissue alterations are present in TKO p53 −/− mice: Prominent inclusions in the nucleus (yellow arrow), inflammation (green arrow) increase in cells with three nuclei (red arrow) and hyperchromatic nuclei with prominent nucleoli as well as pyknotic nuclei (blue arrow). Five mice per group were analyzed for each experiment, except for FACS (3 mice) and statistical differences were calculated by *t*-test. * denotes *p* < 0.05.

Telomere dysfunction and p53 deficiency, independently, have been shown to affect ploidy under regenerating conditions and we wondered whether increased fructose consumption induced similar changes [47,48,49]. On histological examination, we noticed that the fructose diet induced cellular changes that included an increase in cell and nuclear size, number of nuclei per cell, as well as nuclear abnormalities in TKO mice lacking p53 either on a whole-body level or only in the liver (Figure 6A–E; *n* = 5 per group and data not shown). Interestingly, TKO p53 −/−, but not TKO p53 +/+ hepatocytes were significantly larger, resulting in fewer cells per field-of-view (Figure 6A; *n* = 5 per group; *p* < 0.05). The cell size variability is dependent on increased fructose consumption and p53 status as TKO p53 −/− mice on regular chow or TKO p53 +/+ on a fructose diet do not show any significant changes in cell size (Figure 6B; *n* = 5 per group; *p* < 0.05). The cell size changes were accompanied by an increased number of nuclei per cell with a significant increase of hepatocytes carrying three nuclei and, on occasion, four nuclei (Figure 6C; *n* = 5 per group; *p* < 0.05). FACS analysis demonstrated a significant increase in polyploidy in TKO p53 −/− mice (Figure 6D; *n* = 5 per group; *p* < 0.05). Significant abnormalities in the structure of nuclei were evident in TKO p53 −/− hepatocytes based on the presence of enlarged nucleoli, presence of inclusion bodies, and irregular shape (Figure 6B; *n* = 5 per group). While these cellular alterations are compatible with dysplastic changes, no frank carcinoma was observed, indicating that additional steps are required for full transformation.

## 3. Discussion

Together, these studies demonstrate that livers with short telomeres are susceptible to a fructose-rich diet. This susceptibility is reflected in the pronounced activation of p53, increased cell death, and senescence. While the underlying mechanisms of fructose-induced p53 activation in the context of short telomeres remain to be established, fructose has been demonstrated to drive ATP depletion, mitochondrial dysfunction, and elevate reactive oxygen species, all of which either alone or in combination could account for the pronounced p53 activation [50,51]. Given our previous reports that telomere shortening causes significant impairment of mitochondrial function, it is possible that fructose further exacerbates these defects and causes cell death. The increase of p53 in mice with short telomeres is associated with a blunted transcriptional activation of genes driving TG synthesis compared to WT mice. A similar metabolic maladaptation has been reported for TERT deficient mice subjected to a liquid high fat diet as theses mice show decreased expression of many metabolic genes [52]. This contrasts with comparable expression of these genes in TKO mice on a regular chow, suggesting that TKO mice fail to mount an adequate transcriptional in response to increased metabolic load. This maladaptation is also uncovered in aged TKO mice that are fed a regular chow diet, indicating that this maladaptation becomes worse over time [53]. Our studies demonstrate that p53 is a central mediator of the repressed metabolic response in TKO mice. This is supported by the observation that expression levels of genes involved in TG and glycogen synthesis are significantly increased when p53 is deleted. Indeed, the repressive function of p53 is telomere length independent as p53 deficient mice with intact telomeres also respond with increased TG accumulation. These studies indicate a fundamental role of p53 in sugar and lipid metabolism and are in line with previous in vivo studies in p53 deficient mice [46,54]. While our studies in global and liver specific p53 deficient mice support the notion that p53 represses hepatic lipid accumulation in response to fructose, previous studies in germline and liver specific p53 deficient mice have demonstrated a similar repressive function of p53 when mice are fed a HFD [46]. The underlying mechanisms that mediate the observed p53 repression remain to be elucidated when telomeres are short but could be secondary to elevated levels of p63, or direct repression of Shrebp1c [54,55]. While the absence of p53 increased liver fat content in mice with and without short telomeres, our studies demonstrate that short telomeres cause profound histological and cytological abnormalities when p53 is deleted. First, hepatocytes with p53 deletion are susceptible to cell death, which contrasts with low cell death rate in WT mice fed a fructose-rich diet. The increase in cell death is accompanied by significant fibrosis in mice with short telomeres and is dependent on fructose as TKO mice on a regular chow diet have normal liver. Secondly, p53 deficiency leads to severe cytological changes including increased nuclearity, ploidy, and dysplastic changes. These changes are telomere length and fructose dependent as p53 deficient mice (with intact telomeres) on a fructose diet or TKO mice on regular chow diet do not show these cytological alterations. Telomere dysfunction and generalized DNA damage in conjunction with p53 deficiency has been shown to induce polyploidy in proliferating mouse and human cells through endoreplication in an ATM and/or ATR-dependent manner [47,48,56,57]. In the quiescent liver, telomere deprotection is well tolerated in the presence of p53 and does not induce ploidy changes [58]. In contrast, telomere dysfunction causes endoreduplication and polyploidy in the regenerating liver [58]. The regenerating liver undergoes polyploidization in p53 deficient mice, underscoring the fundamental role of short telomeres and p53 for polyploidy under proliferative conditions [58,59]. Our studies extend these observations and suggest that metabolic stress—such as caused by increased fructose consumption—can cause polyploidization when telomeres are dysfunctional and p53 is inactivated. While the mechanisms that underlie this fructose-induced polyploidization remain to be established, studies in plants have shown that metabolic demands can drive endoreduplication and increase ploidy status. Apart from this endogenous, potential metabolite-driven mechanism for polyploidization, the increase in ploidy could be the result of incomplete, mitosis-bypassing cell proliferation in response to increased cell death. Interestingly, our studies indicate a substantial increase of hepatocytes with increased number of nuclei, which could stem from either failed cytokinesis or—less likely—increased cell fusion. An important aspect of the cellular changes is the occurrence of dysplastic changes, including dysmorphic nuclei with inclusions of unclear nature, hyperchromatic changes, and prominent nucleoli. The presence of hepatocytes with these preneoplastic changes could serve as a pool for cells undergoing full malignant transformation. Our findings are of potential relevance for human disease. Of note, telomere shortening has been described to occur in patients with fatty liver disease and increased polyploidy has been described in animal models of NAFLD and NASH and in patients with NASH. Polyploid cells can induce aneuploidy via chromosome missegregation and thereby drive cancer formation [60]. The absence of p53 can further facilitate polyploidy and aneuploidy and predispose to malignant transformation in human cells, as demonstrated by the predisposition of polyploid p53-null mammary cells for malignant transformation compared to their diploid counterparts [61].

Together, our studies indicate that increased fructose consumption induces cellular stress in livers with short telomeres and potently activates a p53 response that represses the transcriptional response to fructose and curtails the accumulation of hepatic fat. Deletion of p53 in the setting of short telomeres increases TG content and induces polyploidy and dysplastic nuclear changes in response to increased fructose consumption. These studies reveal how short telomeres and p53 potently modify fructose metabolism that is potentially relevant for metabolic liver disease and hepatocellular carcinoma.

## 4. Materials and Methods

### 4.1. Mice

Animal experiments were carried out in accordance with the procedures laid out in animal protocol AN5885 approved by the Institutional Animal Care and Use Committee at Baylor. Mice were housed in a standard barrier facility with 12-hour light-dark cycles and received a standard rodent chow diet (PicoLab, 5V5R). All studies were performed with age-and sex-matched mice and if not otherwise stated, male mice between 8 and 16 weeks of age were used. All mice in this study are on a C57/B6 background.

#### 4.1.1. TERT Deficient Mice

Mice deficient for TERT were provided by Ronald A. DePinho [40]. To produce mice with increasing telomere shortening (G1-G3), TERT heterozygous mice were continuously interbred as described [40]. G3 mice are referred as TKO mice and used for all studies, unless stated otherwise.

#### 4.1.2. TERT/p53 −/− Double Mutant Mice

p53 germline deficient mice [62] were purchased from JAX laboratory and crossed to heterozygous TERT mice and compound heterozygous mice were interbred to generate mice with decreasing telomere length that lack or have intact p53.

#### 4.1.3. TERT/p53 fl/fl/AlbCre Mice

Conditional p53 [63] and AlbCre [64] transgenic mice (JAX Laboratory) were crossed to TERT heterozygous mice to generate mice with progressive telomere shortening and p53 fl/fl mice either positive or negative for the Alb-Cre transgene.

Genotyping: Tail snippets were lyzed in 100 mM NaCl, 10 mM Tris (pH 8.0), 1 mM EDTA, 1% SDS, and 1 mg/mL proteinase K and used for the identification of TERT, germline p53, conditional p53, and AlbCre alleles by PCR, as previously described [41].

### 4.2. AAV Experiments to Generate Liver Specific p53 Null Mice

Recombinant AAV-Cre (AAV8.TBG.PI.Cre.rBG, UPenn, Penn Vector Core) was delivered via tail-vein injection at a dose of 2 × 10^11^ GC (genome copies) in a final volume of 200 μL of PBS. Two weeks later, the mice were started on a fructose diet.

### 4.3. Fructose Diet

Mice had free access either to tap water or 30% fructose water for three months, if not indicated otherwise. Body weight, food consumption, and amount of consumed water was documented once weekly throughout the treatment period. 

### 4.4. CLAMS Analysis

Comprehensive Lab Animal Monitoring System (CLAMS) at the Metabolic Core at Baylor was used to measure energy expenditure, food, water intake, and activity.

### 4.5. Assays for Liver Function

Liver function assays were performed by the Laboratory of Comparative Pathology at Baylor College of Medicine. 

### 4.6. RNA Isolation, cDNA Synthesis, and Real-Time PCR

Total liver RNA was extracted with Trizol reagent (Thermo Fisher Scientific). RNA was then digested with DNase I (New Engeland Biolabs) and further cleaned up with RNeasy Mini columns (QIAGEN). cDNA synthesis was carried out with Protoscript II (New England Biolabs) using 0.5 μg of total RNA.

To quantify transcript levels, SensiFAST Probes or SensiFAST SYBR kit (BIOLINE) was used. The primer sequences used for qPCR are listed in Table 1. The ΔΔ CT method was used to calculate the expression levels of the transcripts.

### 4.7. Immunoblotting

Liver lysates were prepared in RIPA buffer (50 mM Tris-HCl, pH7.4, 150 mM NaCl, 0.1% SDS, 0.5% sodium deoxycholate, 1.0% Triton X-100) containing protease (Roche Applied Science) and phosphatase inhibitors (GenDEPOT). Lysates were briefly sonicated and the protein concentration was determined from the centrifuged by Bradford assay. Equal amounts of protein was loaded onto SDS-PAGE gels, and transferred to nitrocellulose membranes (Bio-Rad). Membranes were blocked ((5% skim milk in TBST (50 mM Tris-Cl, pH7.5, 150 mM NaCl, 0.05% Tween 20)) for 1 h and incubated with primary antibodies overnight at 4 °C. All primary antibodies used for these studies are listed in Table 2. Subsequently, membranes were incubated with horseradish peroxidase-coupled anti-mouse or anti-rabbit secondary antibodies (ThermoFisher Scientific, Waltham, MA, USA) and developed with Super Signal West Pico chemiluminescent substrate (ThermoFisher Scientific, Waltham, MA, USA). 

### 4.8. Histology and Immunohistochemistry

Livers were removed, washed with PBS, cut open, and fixed in 4% paraformaldehyde overnight at 4 °C. Tissues were embedded in paraffin, sectioned at 4 µm, and stained with Hematoxylin and Eosin (H&E) for histopathological examination. For immunohistochemistry, tissue sections were deparaffinized and rehydrated in an ethanol series. They were blocked with 3% H_2_O_2_ for 15 min at room temperature. For the biotin- based staining, sections were washed, then blocked with Avidin/Biotin blocking solutions (Vector lab, Burlingame, CA, USA), and blocked with 5% goat serum for 1 h at room temperature. Sections were incubated overnight at 4 °C with the primary antibodies listed in Table 2. They were then washed and incubated for 1 h at room temperature with goat α-rabbit biotinylated secondary antibody (Vector Labs). The sections were developed with a DAB substrate kit (Vector lab) at room temperature, counterstained with haematoxylin and after drying were mounted with Permount mounting medium (Electron Microscopy Sciences, Hatfield, PA, USA). 

Apoptosis: Apoptotic liver cells were identified with the ApopTag^®^ Peroxidase In Situ Apoptosis Detection Kit (Millipore, Burlington, MA, USA) or by cleaved caspase 3 (Cell Signaling, Danvers, MA, USA) staining.

Oil Red O (ORO) and Periodic Acid Schiff (PAS) staining: ORO and PAS staining were carried out and quantified as previously described [65,66].

BrdU staining: Mice were injected with 100 mg/kg BrdU (Amersham Biosciences, Piscataway, NJ, USA) intraperitoneally for two hours. BrdU-positive cells were identified with IHC using an anti-BrdU antibody (Ab3, Thermo Scientific, Fremont, CA, USA).

Senescence: Detection of SA-β-gal activity was performed as described [67]. Briefly, frozen sections of liver tissue were stained overnight with PBS containing 1 mM MgCl_2_, 1 mg/mL X-Gal and 5 mM of each Potassium ferricyanide after fixation in 0.5% Gluteraldehyde and several washes with PBS. Sections were counterstained with Eosin.

### 4.9. Immunofluorescence

Antigen retrieval was carried out on deparaffinized and rehydrated 10 μm liver sections in a microwave for 20 min using TE buffer (10 mM Tris- Cl, pH 8.0, 1 mM EDTA, 0.05% Tween 20). Slides were then treated with 3% H_2_O_2_ for 15 min (room temperature), blocked with 2.5% horse serum (1 h at room temperature) and incubated overnight with antibodies recognizing p21 and cleaved caspase 3 (see Table 2) at 1:500 at 4 °C. Slides were washed with PBS and then incubated with Alexa Fluor 488 conjugated anti-mouse/rabbit IgG secondary antibodies (Thermo Fisher Scientific) at room temperature for 1 h. After washing in PBS, the slides were mounted with anti-fade mounting media. Positive cells were counted in 10 high power-fields (HPF) and the results were reported as positive cells per HPF.

### 4.10. Fibrosis Studies

#### 4.10.1. Sirius Red

Fixed (10% formalin) 10 μm liver sections were deparaffinized and rehydrated and stained according to instructions provided by the picrosirius red staining kit (Polysciences, Inc., Warrington, PA, USA). Briefly, sections were stained for two minutes in phosphomolybdic acid and stained with picrosirius red for one hour, followed by two minutes in acidified water and 45 s in 70% ethanol. Subsequently, the slides were dehydrated in ethanol and cleared in xylene. Slides were then mounted in Permount medium. The quantification of fibrosis was performed using ImageJ (NIH) on ten random low-power (40×) images. The results are reported as percent fibrotic area.

#### 4.10.2. Hepatic Hydroxyproline Content

The Total Collagen Assay (QuickZyme) was used according to the manufacturer’s instructions and as described recently to determine hepatic hydroxyproline content [68,69]. Results are expressed as micrograms hydroxyproline per gram liver tissue.

### 4.11. Ploidy Determination

To determine DNA content (ploidy), hepatocytes were isolated by collagenase perfusion as described previously [70]. Hepatocytes were then stained with propidium iodide at a concentration of 50 μg/mL) in 0.2% Tween-PBS supplemented with 1 μg/mL RNAse A (Molecular Probes) after fixation in 80% ice-cold ethanol. Cells were analyzed on a FACSCalibur. Statistical analysis was performed by using the CellQuest software (BD Biosciences).

### 4.12. Statistics

GraphPad Prism (GraphPad Software, La Jolla, CA, USA) was used for statistical analysis and results are reported as mean values ± s.e.m. Student’s *t*-test was used to determine the statistical differences with P-values less than 0.05 considered statistically significant.

## Figures and Tables

**Table 1:** Sequences of primer sequences used for RT-qPCR.

Primer	Sequence
Bax Forward	CAGGATGCGTCCACCAAGAA
Bax Reverse	AGTCCGTGTCCACGTCAGCA
Puma Forward	ACGACCTCAACGCGCAGTACG
Puma Reverse	GAGGAGTCCCATGAAGAGATTG
p21 Forward	AGATCCACAGCGATATCCAGAC
p21 Reverse	ACCGAAGAGACAACGGCACACT
Gys2 Forward	CCTGGGCAGATATTACCAGCAT
Gys2 Reverse	TTCCCCTTTGGAAAGTGGTTCA
Gsk3b Forward	CAGCAGCCTTCAGCTTTTGG
Gsk3b Reverse	AGCTCTCGGTTCTTAAATCGCT
Pygl Forward	AACACTATGCGCCTCTGGTC
Pygl Reverse	GCTGGATGGCTACCTGATCT
mSCD1 Forward	CCGGAGACCCCTTAGATCGA
mSCD1 Reverse	TAGCCTGTAAAAGATTTCTGCAAACC
mACC1 Forward	TGACAGACTGATCGCAGAGAAAG
mACC1 Reverse	TGGAGAGCCCCACACACA
mSREBP-1c Forward	GGAGCCATGGATTGCACATT
mSREBP-1c Reverse	GGCCCGGGAAGTCACTGT
mFASN Forward	GCTGCGGAAACTTCAGGAAAT
mFASN Reverse	AGAGACGTGTCACTCCTGGACTT
beta Actin Forward	CCCTGTATGCCTCTGGTCGTACCAC
beta Actin Reverse	GCCAGCCAGGTCCAGACGCAGGATG

**Table 2:** Listed antibodies used in this study.

Antibody	Vendor	Cat Number	Dilution
Recombinant Anti-p21 antibody [EPR18021]	Abcam	ab188224	1:1000 (IHC/WB)
Phospho-p53 (Ser15) Antibody	Cell Signaling	#9284	1:100 (WB)
Cleaved Caspase-3 Antibody (Asp175) (D3E9)	Cell Signaling	#9579	1:250 (IHC)

## Data Availability

All data, tables and figures are original and have not been published anywhere else.

## Supplementary Materials

The following are available online at https://www.mdpi.com/article/10.3390/metabo11060394/s1, Figure S1: TKO livers are indistinguishable from WT mice on a regular chow diet, Figure S2: Mice with short telomeres accumulate less fat in liver tissue upon fructose treatment, Figure S3: P53 deletion increases fat accumulation in TKO mice.

## References

[1] Blackburn E.H., Gall J.G. (1978). A tandemly repeated sequence at the termini of the extrachromosomal ribosomal RNA genes in Tetrahymena. J. Mol. Biol..

[2] Szostak J.W., Blackburn E.H. (1982). Cloning yeast telomeres on linear plasmid vectors. Cell.

[3] Greider C., Blackburn E.H. (1985). Identification of a specific telomere terminal transferase activity in tetrahymena extracts. Cell.

[4] Greider C., Blackburn E.H. (1987). The telomere terminal transferase of tetrahymena is a ribonucleoprotein enzyme with two kinds of primer specificity. Cell.

[5] Greider C., Blackburn E.H. (1989). A telomeric sequence in the RNA of Tetrahymena telomerase required for telomere repeat synthesis. Nat. Cell Biol..

[6] Harley C.B., Futcher A.B., Greider C. (1990). Telomeres shorten during ageing of human fibroblasts. Nat. Cell Biol..

[7] Hastie N.D., Dempster M., Dunlop M.G., Thompson A.M., Green D.K., Allshire R.C. (1990). Telomere reduction in human colorectal carcinoma and with ageing. Nat. Cell Biol..

[8] Takai H., Smogorzewska A., de Lange T. (2003). DNA Damage Foci at Dysfunctional Telomeres. Curr. Biol..

[9] di Fagagna F.D., Reaper P.M., Clay-Farrace L., Fiegler H., Carr P., von Zglinicki T., Saretzki G., Carter N.P., Jackson S.P. (2003). A DNA damage checkpoint response in telomere-initiated senescence. Nature.

[10] Bodnar A.G., Ouellette M., Frolkis M., Holt S.E., Chiu C.-P., Morin G., Harley C.B., Shay J.W., Lichtsteiner S., Wright W.E. (1998). Extension of Life-Span by Introduction of Telomerase into Normal Human Cells. Science.

[11] Chin L., Artandi S.E., Shen Q., Tam A., Lee S.-L., Gottlieb G.J., Greider C.W., DePinho R.A. (1999). p53 deficiency rescues the adverse effects of telomere loss and cooperates with telomere dysfunction to acceler-ate carcinogenesis. Cell.

[12] Herbig U., Ajobling W., Chen B.P., Chen D.J., Sedivy J.M. (2004). Telomere Shortening Triggers Senescence of Human Cells through a Pathway Involving ATM, p53, and p21CIP1, but Not p16INK4a. Mol. Cell.

[13] Armanios M., Blackburn E.H. (2012). The telomere syndromes. Nat. Rev. Genet..

[14] Sahin E., Depinho R.A. (2010). Linking functional decline of telomeres, mitochondria and stem cells during ageing. Nat. Cell Biol..

[15] Donate L.E., Blasco M.A. (2011). Telomeres in cancer and ageing. Philos. Trans. R. Soc. B.

[16] O’Sullivan J.N., Bronner M.P., Brentnall T.A., Finley J.C., Shen W.-T., Emerson S., Emond M.J., Gollahon K.A., Moskovitz A.H., Crispin D.A. (2002). Chromosomal instability in ulcerative colitis is related to telomere shortening. Nat. Genet..

[17] Risques R.A., Rabinovitch P.S., Brentnall T.A. (2006). Cancer surveillance in inflammatory bowel disease: New molecular approaches. Curr. Opin. Gastroenterol..

[18] Risques R.A., Lai L.A., Brentnall T.A., Li L., Feng Z., Gallaher J., Mandelson M.T., Potter J.D., Bronner M.P., Rabinovitch P.S. (2008). Ulcerative Colitis Is a Disease of Accelerated Colon Aging: Evidence From Telomere Attrition and DNA Damage. Gastroenterology.

[19] Vallejo A.N. (2004). T-cell senescence: A culprit of immune abnormalities in chronic inflammation and persistent infection. Trends Mol. Med..

[20] Armanios M. (2012). Telomerase and idiopathic pulmonary fibrosis. Mutat. Res..

[21] Armanios M. (2009). Syndromes of Telomere Shortening. Annu. Rev. Genom. Hum. Genet..

[22] Calado R.T., Young N.S. (2009). Telomere Diseases. N. Engl. J. Med..

[23] Garcia C.K., Wright W.E., Shay J.W. (2007). Human diseases of telomerase dysfunction: Insights into tissue aging. Nucleic Acids Res..

[24] Choudhury A.R., Ju Z., Djojosubroto M.W., Schienke A., Lechel A., Schaetzlein S., Jiang H., Stepczynska A., Wang C., Buer J. (2006). Cdkn1a deletion improves stem cell function and lifespan of mice with dysfunctional telomeres without accelerating cancer formation. Nat. Genet..

[25] Sperka T., Wang J., Rudolph K.L. (2012). DNA damage checkpoints in stem cells, ageing and cancer. Nat. Rev. Mol. Cell Biol..

[26] Savage S.A., Bertuch A.A. (2010). The genetics and clinical manifestations of telomere biology disorders. Genet. Med..

[27] Savage S.A., Dokal I., Armanios M., Aubert G., Cowen E.W., Domingo D.L., Giri N., Greene M.H., Orchard P.J., Tolar J. (2009). Dyskeratosis congenita: The first NIH clinical research workshop. Pediatr. Blood Cancer.

[28] Iredale J. (2008). Defining therapeutic targets for liver fibrosis: Exploiting the biology of inflammation and repair. Pharmacol. Res..

[29] Savage S.A. (2014). Human Telomeres and Telomere Biology Disorders. Prog. Mol. Biol. Transl. Sci..

[30] Sheka A.C., Adeyi O., Thompson J., Hameed B., Crawford P.A., Ikramuddin S. (2020). Nonalcoholic Steatohepatitis. JAMA.

[31] Younossi Z., Anstee Q.M., Marietti M., Hardy T., Henry L., Eslam M., George J., Bugianesi E. (2018). Global burden of NAFLD and NASH: Trends, predictions, risk factors and prevention. Nat. Rev. Gastroenterol. Hepatol..

[32] Bray G.A., Popkin B. (2014). Dietary Sugar and Body Weight: Have We Reached a Crisis in the Epidemic of Obesity and Diabetes?. Diabetes Care.

[33] Abdelmalek M.F., Suzuki A., Guy C.D., Unalp-Arida A., Colvin R., Johnson R.J., Diehl A.M. (2010). For the Nonalcoholic Steatohepatitis Clinical Research Network Increased fructose consumption is associated with fibrosis severity in patients with nonalcoholic fatty liver disease. Hepatology.

[34] Spruss A., Kanuri G., Stahl C., Bischoff S.C., Bergheim I. (2012). Metformin protects against the development of fructose-induced steatosis in mice: Role of the intestinal barrier function. Lab. Investig..

[35] Sellmann C., Priebs J., Landmann M., Degen C., Engstler A.J., Jin C.J., Gärttner S., Spruss A., Huber O., Bergheim I. (2015). Diets rich in fructose, fat or fructose and fat alter intestinal barrier function and lead to the development of nonalcoholic fatty liver disease over time. J. Nutr. Biochem..

[36] Basaranoglu G. (2013). Fructose as a key player in the development of fatty liver disease. World J. Gastroenterol..

[37] Stanhope K.L., Schwarz J.M., Keim N.L., Griffen S.C., Bremer A.A., Graham J., Hatcher B., Cox C.L., Dyachenko A., Zhang W. (2009). Consuming fructose-sweetened, not glucose-sweetened, beverages increases visceral adiposity and lipids and decreases insulin sensitivity in overweight/obese humans. J. Clin. Investig..

[38] Federico A., Rosato V., Masarone M., Torre P., Dallio M., Romeo M., Persico M. (2021). The Role of Fructose in Non-Alcoholic Steatohepatitis: Old Relationship and New Insights. Nutrition.

[39] Samuel V.T. (2011). Fructose induced lipogenesis: From sugar to fat to insulin resistance. Trends Endocrinol. Metab..

[40] Sahin E., Colla S., Liesa M., Moslehi J., Müller F.L., Guo M., Cooper M., Kotton D.N., Fabian A.J., Walkley C. (2011). Telomere dysfunction induces metabolic and mitochondrial compromise. Nature.

[41] Amano H., Chaudhury A., Rodriguez-Aguayo C., Lu L., Akhanov V., Catic A., Popov Y.V., Verdin E., Johnson H., Stossi F. (2019). Telomere Dysfunction Induces Sirtuin Repression that Drives Telomere-Dependent Disease. Cell Metab..

[42] Dong K., Zhang Y., Huang J.-J., Xia S.-S., Yang Y. (2020). Shorter leucocyte telomere length as a potential biomarker for nonalcoholic fatty liver disease-related advanced fibrosis in T2DM patients. Ann. Transl. Med..

[43] Lechel A., Manns M.P., Rudolph K.L. (2004). Telomeres and telomerase: New targets for the treatment of liver cirrhosis and hepatocellular carcinoma. J. Hepatol..

[44] Farazi P.A., Glickman J., Horner J., Depinho R.A. (2006). Cooperative Interactions of p53 Mutation, Telomere Dysfunction, and Chronic Liver Damage in Hepatocellular Carcinoma Progression. Cancer Res..

[45] Jiang P., Du W., Wang X., Mancuso A., Gao X., Wu M., Yang X. (2011). p53 regulates biosynthesis through direct inactivation of glucose-6-phosphate dehydrogenase. Nat. Cell Biol..

[46] Wang X., Zhao X., Gao X., Mei Y., Wu M. (2012). A new role of p53 in regulating lipid metabolism. J. Mol. Cell Biol..

[47] Davoli T., de Lange T. (2011). The causes and consequences of polyploidy in normal development and cancer. Annu. Rev. Cell Dev. Biol..

[48] Davoli T., Denchi E.L., de Lange T. (2010). Persistent Telomere Damage Induces Bypass of Mitosis and Tetraploidy. Cell.

[49] Sheahan S., Bellamy C.O., Treanor L., Harrison D.J., Prost S. (2003). Additive effect of p53, p21 and Rb deletion in triple knockout primary hepatocytes. Oncogene.

[50] Lim J.S., Mietus-Snyder M., Valente A., Schwarz J.-M., Lustig R.H. (2010). The role of fructose in the pathogenesis of NAFLD and the metabolic syndrome. Nat. Rev. Gastroenterol. Hepatol..

[51] Labuschagne C.F., Zani F., Vousden K.H. (2018). Control of metabolism by p53—Cancer and beyond. Biochim. Biophys. Acta Bioenerg..

[52] Alves-Paiva R.M., Kajigaya S., Feng X., Chen J., Desierto M., Wong S., Townsley D.M., Donaires F.S., Bertola A., Gao B. (2017). Telomerase enzyme deficiency promotes metabolic dysfunction in murine hepatocytes upon dietary stress. Liver Int..

[53] Missios P., Zhou Y., Guachalla L.M., Von Figura G., Wegner A., Chakkarappan S.R., Binz T., Gompf A., Hartleben G., Burkhalter M. (2014). Glucose substitution prolongs maintenance of energy homeostasis and lifespan of telomere dysfunctional mice. Nat. Commun..

[54] Porteiro B., Fondevila M.F., Delgado T.C., Iglesias C., Imbernon M., Iruzubieta P., Crespo J., Zabala-Letona A., Fernø J., González-Terán B. (2017). Hepatic p63 regulates steatosis via IKKβ/ER stress. Nat. Commun..

[55] Yahagi N., Shimano H., Matsuzaka T., Najima Y., Sekiya M., Nakagawa Y., Ide T., Tomita S., Okazaki H., Tamura Y. (2003). p53 Activation in Adipocytes of Obese Mice. J. Biol. Chem..

[56] Hockemeyer D., Daniels J.-P., Takai H., De Lange T. (2006). Recent Expansion of the Telomeric Complex in Rodents: Two Distinct POT1 Proteins Protect Mouse Telomeres. Cell.

[57] Kibe T., Osawa G.A., Keegan C.E., de Lange T. (2010). Telomere Protection by TPP1 Is Mediated by POT1a and POT1b. Mol. Cell. Biol..

[58] Denchi E.L., Celli G., De Lange T. (2006). Hepatocytes with extensive telomere deprotection and fusion remain viable and regenerate liver mass through endoreduplication. Genes Dev..

[59] Kurinna S., Stratton S.A., Coban Z., Schumacher J.M., Grompe M., Duncan A.W., Barton M.C. (2013). p53 regulates a mitotic transcription program and determines ploidy in normal mouse liver. Hepatology.

[60] Sansregret L., Vanhaesebroeck B., Swanton C. (2018). Determinants and clinical implications of chromosomal instability in cancer. Nat. Rev. Clin. Oncol..

[61] Fujiwara T., Bandi M., Nitta M., Ivanova E.V., Bronson R.T., Pellman D. (2005). Cytokinesis failure generating tetraploids promotes tumorigenesis in p53-null cells. Nat. Cell Biol..

[62] Jacks T., Remington L., Williams B., Schmitt E.M., Halachmi S., Bronson R.T., Weinberg R.A. (1994). Tumor spectrum analysis in p53-mutant mice. Curr. Biol..

[63] Marino S., Vooijs M., Van Der Gulden H., Jonkers J., Berns A. (2000). Induction of medulloblastomas in p53-null mutant mice by somatic inactivation of Rb in the external granular layer cells of the cerebellum. Genes Dev..

[64] Postic C., Shiota M., Niswender K.D., Jetton T.L., Chen Y., Moates J.M., Shelton K.D., Lindner J., Cherrington A.D., Magnuson M.A. (1999). Dual roles for glucokinase in glucose homeostasis as determined by liver and pancreatic beta cell-specific gene knock-outs using Cre recombinase. J. Biol Chem..

[65] Mehlem A., Hagberg C.E., Muhl L., Eriksson U., Falkevall A. (2013). Imaging of neutral lipids by oil red O for analyzing the metabolic status in health and disease. Nat. Protoc..

[66] Saitoh Y., Terada N., Saitoh S., Ohno N., Fujii Y., Ohno S. (2010). Histochemical approach of cryobiopsy for glycogen distribution in living mouse livers under fasting and local circulation loss conditions. Histochem. Cell Biol..

[67] Serrano M., Lin A.W., McCurrach M.E., Beach D., Lowe S.W. (1997). Oncogenic ras Provokes Premature Cell Senescence Associated with Accumulation of p53 and p16INK4a. Cell.

[68] MacKenzie B., Henneke I., Hezel S., Al Alam D., El Agha E., Chao C.-M., Quantius J., Wilhelm J., Jones M., Goth K. (2015). Attenuating endogenous Fgfr2b ligands during bleomycin-induced lung fibrosis does not compromise murine lung repair. Am. J. Physiol. Cell. Mol. Physiol..

[69] Loyer X., Paradis V., Hénique C., Vion A.-C., Colnot N., Guerin C.L., Devue C., On S., Scetbun J., Romain M. (2016). Liver microRNA-21 is overexpressed in non-alcoholic steatohepatitis and contributes to the disease in experimental models by inhibiting PPARα expression. Gut.

[70] Overturf K., Al-Dhalimy M., Tanguay R., Brantly M., Ou C.-N., Finegold M., Grompe M. (1996). Hepatocytes corrected by gene therapy are selected in vivo in a murine model of hereditary tyrosinaemia type I. Nat. Genet..

