# Supplementary material for: Fructose Causes Liver Damage, Polyploidy, and Dysplasia in the Setting of Short Telomeres and p53 Loss
Source: Metabolites. 2021 Jun 17;11(6):394. doi: 10.3390/metabo11060394 (PMC8234056; doi:10.3390/metabo11060394)
Supplement: Supplementary file 1 [file metabolites-11-00394-s001.zip › metabolites-1243512-supplementary.pdf]

metabolites

Article

Fructose Causes Liver Damage, Polyploidy and Dysplasia in the Setting of Short Telomeres and p53 Loss

Christopher Chronowski [1], Viktor Akhanov [1], Doug Chan [2], Andre Catic [1,2], Milton Finegold [4] and Ergün Sahin [1,3,*]

[1] Huffington Center on Aging, Baylor College of Medicine, Houston, TX 77030, USA; christopher.chronowski@bcm.edu
[2] Department of Molecular and Cellular Biology, Baylor College of Medicine, Houston, TX 77030, USA;
[3] Department of Physiology and Biophysics, Baylor College of Medicine, Houston, 77030TX, USA;
[4] Department of Pathology, Baylor College of Medicine, Houston, TX 77030, USA;
* Correspondence: esahin@bcm.edu; Tel.: 713-798-6685; Fax.: 713-798-4146

Supplementary Materials

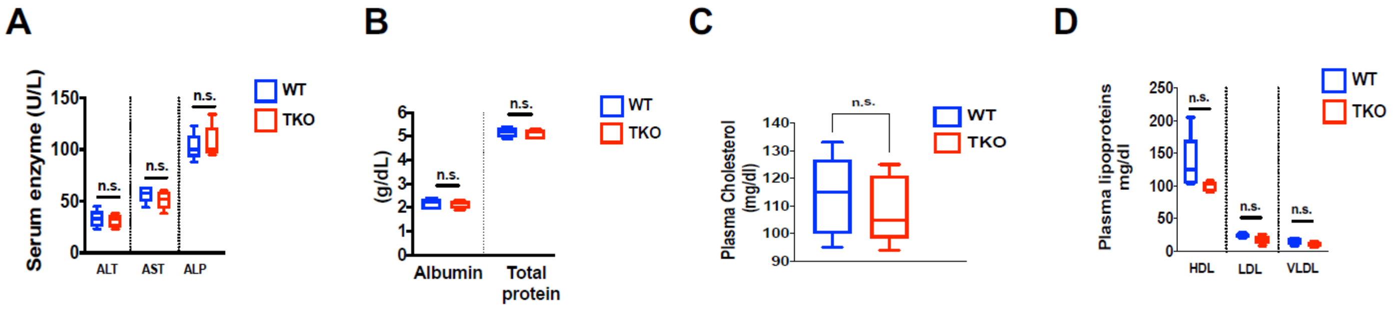

Figure S1. TKO livers are indistinguishable from WT mice on regular chow diet (A–D)Transaminases, albumin, globulin, cholesterol, HLD, LDL and VLDL are in normal range in TKO mice indicating hepatocytes are not damaged and have normal synthesis capacity. 5 mice were analyzed per group for each experiment and statistical differences were calculated by t-test. n.s. = not significant.

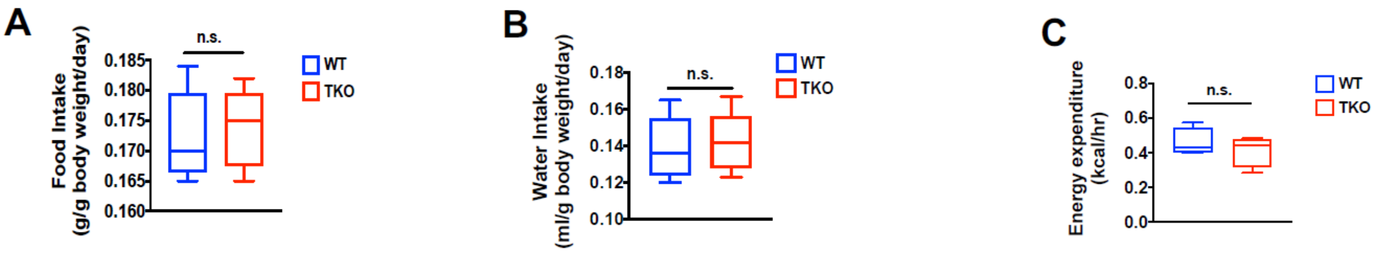

Figure S2. Mice with short telomeres accumulate less fat in liver tissue upon fructose treatment (A–C) No differences in food, water intake and energy expenditure between WT and TKO mice on a fructose diet. 5 mice per group were analyzed analyzed for each experiment and statistical differences were calculated by t-test. * denotes $p < 0.05$.

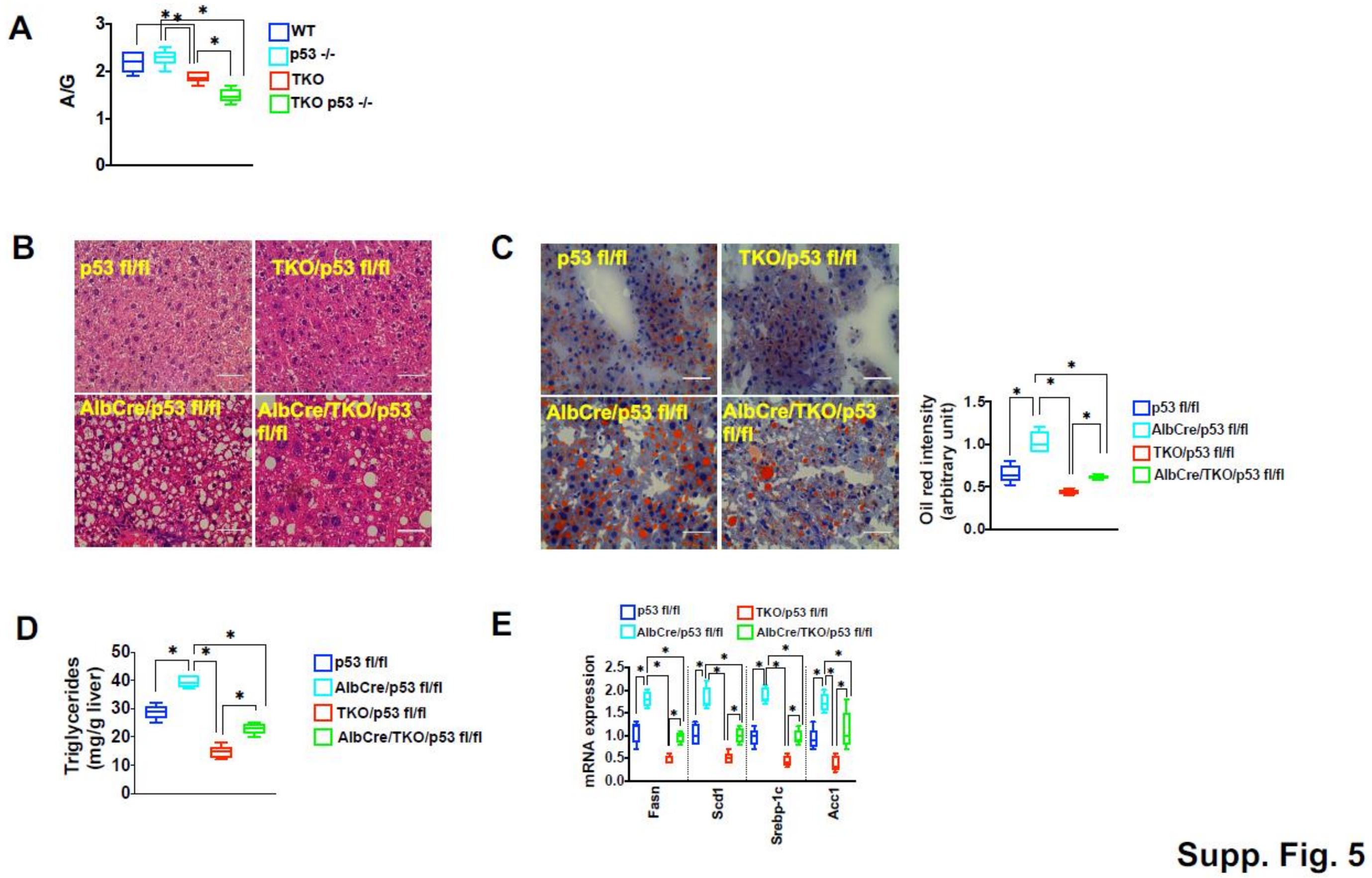

Figure S3. p53 deletion increases fat accumulation in TKO mice (A) Albumin/Globulin ratio is further reduced in TKO p53 -/- mice indicating further compromise in TKO liver synthesis capacity compared to TKO mice with intact p53 (B) H&E staining shows increase in vacuole size in TKO p53 -/- mice suggesting increased fat (C) p53 deficiency in TKO livers alone is sufficient to increase fat content as determined by ORO staining (D) TG content in TKO liver-specific p53 null mice (AlbCre/TKO/p53 fl/fl) is significantly incresed compared to TKO mice with intact p53 (E) RT-qPCR analysis demonstrates increased expression of TG synthesisregulating genes in AlbCre/TKO/p53 fl/fl mice. 5-8 mice per group were analyzed analyzed for each experiment and statistical differences were calculated by t-test. * denotes $p < 0.05$.
